# Quad test for fetal aneuploidy screening as a predictor of small-for-gestational age fetuses: a population-based study

**DOI:** 10.1186/s12884-020-03298-9

**Published:** 2020-10-15

**Authors:** Rakchanok Boonpiam, Chanane Wanapirak, Supatra Sirichotiyakul, Ratanaporn Sekararithi, Kuntharee Traisrisilp, Theera Tongsong

**Affiliations:** Department of Obstetrics and Gynecology, Faculty of Medicine, Chiang Mai University, Chiang Mai, 50200 Thailand

## Abstract

**Background:**

To identify the relationship between quadruple test for aneuploidy screening (alpha-fetoprotein: AFP; free beta-human chorionic gonadotropin: b-hCG; unconjugated estriol: uE3 and inhibin-A: IHA) and fetal growth restriction and to construct predictive models for small-for-gestational-age (SGA) fetuses.

**Methods:**

Women who underwent quadruple test for aneuploidy were followed-up for final outcomes. The multiples of the median (MoMs) of the four biochemical markers for the SGA group and those of normal fetuses were compared. The models for predicting SGA by the individual biomarkers and their combination were constructed using binary logistic regression analysis, and their diagnostic performances in predicting SGA were determined.

**Results:**

Of 10,155 eligible pregnant women, 578 (5.7%) and 9577 (94.3%) had SGA and normal growth, respectively. High levels of AFP, b-hCG and IHA but low levels of uE3 significantly increased the risk of SGA. The constructed predictive equations had predictive performance for SGA, with areas under the receiver-operated characteristic curve of 0.724, 0.655, 0.597, 0.664 and 0.754 for AFP, b-hCG, uE3, IHA, and the combination, respectively.

**Conclusion:**

The quad test for aneuploidy screening could also be used as a predictor of SGA, without extra-effort and extra-cost.

## Background

Small for gestational age (SGA), usually defined as fetuses with birthweight of less than the 10th percentile of the gestational age, is one of the common conditions of high-risk pregnancy, leading to poor pregnancy outcomes, such as perinatal morbidity and mortality as well as abnormal neurodevelopment. Also, it can increase poor maternal outcomes, such as higher rate of cesarean section, maternal depression,higher cost of antenatal and postnatal care, etc. The incidence of SGA in developing countries varies from 6 to 30% of live births [1], depending on the definition and criteria for diagnosis. Recently, many techniques have been developed to achieve early detection of SGA in order to render the care needed by a high-risk pregnancy and provide effective antenatal care, thereby ensuring the prevention of unexpected adverse outcomes for both mothers and infants. To date, there is no well accepted method to predict SGA among low-risk pregnant women; therefore, new effective methods must be sought. Currently, the implementation of serum biomarker screening (quad test) for fetal aneuploidy has commenced worldwide, and many studies have shown that additional information derived from such screenings, other than aneuploidy risk estimation, can also be used to identify the risk of adverse obstetric outcomes, such as fetal growth restriction, preeclampsia and preterm birth [2–8]. However, there is no guideline or recommendation concerning how to apply this useful information in actual practice. Several studies demonstrated an association between individual marker levels and rate of SGA, but most of them had only a limited number of women, and it is difficult to implement these findings in actual practice. Moreover, it is well established that racial factors strongly impact on serum marker levels [9–13], and the normative data of serum biomarkers derived from Caucasian women cannot accurately be interpreted when used in other parts of the world. Though several studies have linked abnormal serum marker levels to higher rate of SGA, no study provides objective methods or models derived from individual serum markers that can be clinically used to determine the probability of SGA. In several developing countries, quad test (serum marker screening) for fetal aneuploidy has been implemented free of charge, with the cost covered by the government. SGA is one of the leading causes of perinatal morbidity and mortality, but many of such adverse outcomes can probably be prevented if high risk fetuses are identified and early detection as well as proper surveillance is provided. Quad test, though primarily aimed at aneuploidy screening, can also provide some useful information in estimating the risk of poor pregnancy outcomes [2, 4, 6–8, 14–17]. In other words, quad test may be helpful in early detection and effective in the management of fetuses at risk of SGA. Therefore, we can probably take advantage of the quad test used in daily practice to estimate the risk of SGA using the same screen without any additional cost and workload. Accordingly, we carried out this study to identify the performance of the second trimester serum marker screening (quad test) in predicting SGA and to develop a predictive model for SGA, like the predictive model for fetal Down syndrome. With the newly created model, we hope that for each blood sample analysis, the serum biomarker machine could report the risk of trisomy 21, trisomy 18, and trisomy 13 and the risk of SGA in the same setting. The main objectives are as follows: 1) To identify the relationship between second trimester serum markers or quadruple (quad) test, consisting of alpha fetoprotein (AFP), free beta-human chorionic gonadotrophin (b-hCG), unconjugated estriol (uE3), and inhibin-A (IHA), and the rate of SGA. 2) In case of a significant relationship, the model predicting the risk of SGA is constructed.

## Methods

A cohort as well as diagnostic study was conducted as a secondary analysis of a prospective database of the maternal-fetal medicine (MFM) unit of Chiang Mai University, Thailand. The database was developed under the “Prenatal Control of Down Syndrome Project”of the National Health Security Office, Thailand. Under the project, all pregnant women that attended our antenatal care clinic and our network hospitals were offered quad test as a screening test for fetal Down syndrome in the second trimester free of charge. This study was conducted with ethical approval by the Institutional Review Board (The Research Ethics Committee 4; Faculty of Medicine, Chiang Mai University; Study Code: OBG-2562-06069 / Research ID: 06069). This diagnostic study was conducted with adherence to the STARD and TRIPOD standards [18, 19]. All the women, who participated in the project, provided written informed consent. The study population was pregnant women who attended antenatal care at Maharaj Nakorn Chiang Mai Hospital and the network hospitals in the northern part of Thailand and underwent second trimester quad test for fetal Down syndrome screening, with known pregnancy outcomes, between January 2016 and October 2019.

### Database development

The primary project was undertaken to assess the efficacy of the maternal quadruple (quad) test in our population in the detection of fetal Down syndrome. All pregnancies were prospectively followed up for the pregnancy outcomes and fetal status of aneuploidy. All participants were of Thai ethnicity and were living in the North of Thailand. They participated in the project with informed consent after counseling by the project team. The baseline characteristics (age, parity, body weight, ethnicity, medical conditions, etc.) and laboratory analysis of the serum biomarkers were reviewed by the authors and prospectively obtained. The serum biomarker levels of all collected samples were determined at the same project laboratory (completely automated assay, DELFIA® Xpress system; Perkin Elmer, Waltham, MA, USA), with the standard immunoassay kits of AFP, b-hCG, uE3, and IHA. The quad screens were conducted free, as they were financially covered by the National Health Security Office, Thailand. The participants were followed-up for obstetric outcomes, such as birthweight, gestational weeks at birth, route of delivery, fetal anomalies, obstetric complications, etc. The newborns were assessed by pediatricians. Chromosome studies were performed only in women categorized as high risk by the quad test or newborns with suspicion of abnormalities after assessment by the neonatologists. The chromosome abnormalities were confirmed either by amniocentesis or cytogenetic studies after birth, whereas diagnoses of normal chromosomes were confirmed by cytogenetic work-up or the conclusion by the neonatologists in cases where cytogenetic study was not done.

### Data retrieval

The project database, which was developed between 2016 and 2019, was accessed to obtain the records meeting the inclusion criteria as well as having complete information of baseline characteristics and obstetric data, such as maternal age and weight, underlying medical diseases, smoking history, gestational week of blood sampling for maternal serum biomarkers, gestational week at birth, baby weight and anomalies. The inclusion criteria for retrieval of the records are as follows: 1) single gestation, 2) underwent serum biomarker test (quad test) at gestational age of 15–21 weeks, and 3) availability of final obstetric outcomes. The cases with the following criteria were excluded: 1) multifetal gestation, 2) fetal anomaly or aneuploidy, 3) unavailability of obstetric outcomes, 4) pregnancy termination before 20 weeks of pregnancy, and 5) significant medical complications, for examples, uncorrected cyanotic heart disease, uncontrolled hyperthyroidism, renal impairment, etc.

### Data processing

The women who met the inclusion criteria were divided into two groups: pregnancies without SGA (control group) and pregnancies with SGA (study group). All records, including baseline characteristics, serum biomarker levels, obstetric and neonatal outcomes, were reviewed and validated. The definitions used in this study are as follows: 1) Gestational age: Gestational age was based on crown-rump length (CRL) in the first trimester or biparietal diameter (BPD) in the second trimester. 2) Second trimester serum screening: Screening test for fetal aneuploidy in the second trimester (quad screen) using four serum biomarkers, including maternal serum AFP, b-hCG, uE3, and IHA, collected at gestational age of 15–21 weeks. Abnormal levels of the biomarkers were defined as the levels of greater than 2 MoM for AFP, b-hCG and IHA and less than 0.5 MoM for uE3 (based on previous studies). 3) Fetal growth restriction: A fetus with birth weight lower than the 10th percentile of the gestational date [20].

### Sample size estimation

Based on previous studies, the relative risk of SGA among pregnant women with abnormal quad screen (elevated AFP) is approximately 1.6–4.0 [7]. To estimate the sample size, a cohort study with an estimated relative risk of 1.8 and a prevalence of SGA in the control group of approximately 7% needs a sample size of at least 536 affected cases, at 95% confidence and 80% power of test.

### Primary outcome

Incidences of SGA among pregnant women with normal and abnormal concentrations of the four serum biochemical markers: AFP, b-hCG, uE3, and IHA.

### Statistical analysis

The statistical procedures were undertaken using the SPSS software (IBM Corp. Released 2012; IBM SPSS Statistics for Windows, Version 21.0. Armonk, New York). The statistical techniques are the same as those used in determining the fetal risk of aneuploidy, summarized as the followings. The multiples of the median (MoMs) of the four biochemical markers (AFP, b-hCG, uE3 and IHA) were obtained by the following steps: (1) performing regression analysis, using a stepwise technique, of the log10 levels of the four serum markers as a dependent variable against potential independent factors, for examples gestational week of blood sampling, maternal age and weight, history of smoking, etc.; (2) determining the predicted log10 levels of each serum marker of quad test for individual women by using the constructed model in Step 1; (3) transforming the obtained log10 from the prior step to the predicted level of the serum markers; (4) calculating the MoMs by dividing the actual measured values of the serum markers of all subjects by their predicted values. The models for estimating the risk of small for gestational age (SGA) were constructed using a binary logistic regression method, with serum markers as dependent parameters and SGA as an independent parameter. Using the adjusted MoMs, the log-Gaussian distributions of each serum marker for SGA was derived. The effectiveness of the created models was determined by receiver-operated characteristic (ROC) curves, together with the sensitivity and false positive rate in the risk estimation for SGA. The effectiveness of each serum marker and their combination was assessed by comparing the ROC area under the curve of the serum markers. The positive likelihood ratio of SGA for individual women was also determined by dividing the sensitivity by the false positive rate of each serum marker. The final risk of SGA was obtained by multiplying the positive likelihood ratio by the background risk.

## Results

**Fig. 1:**
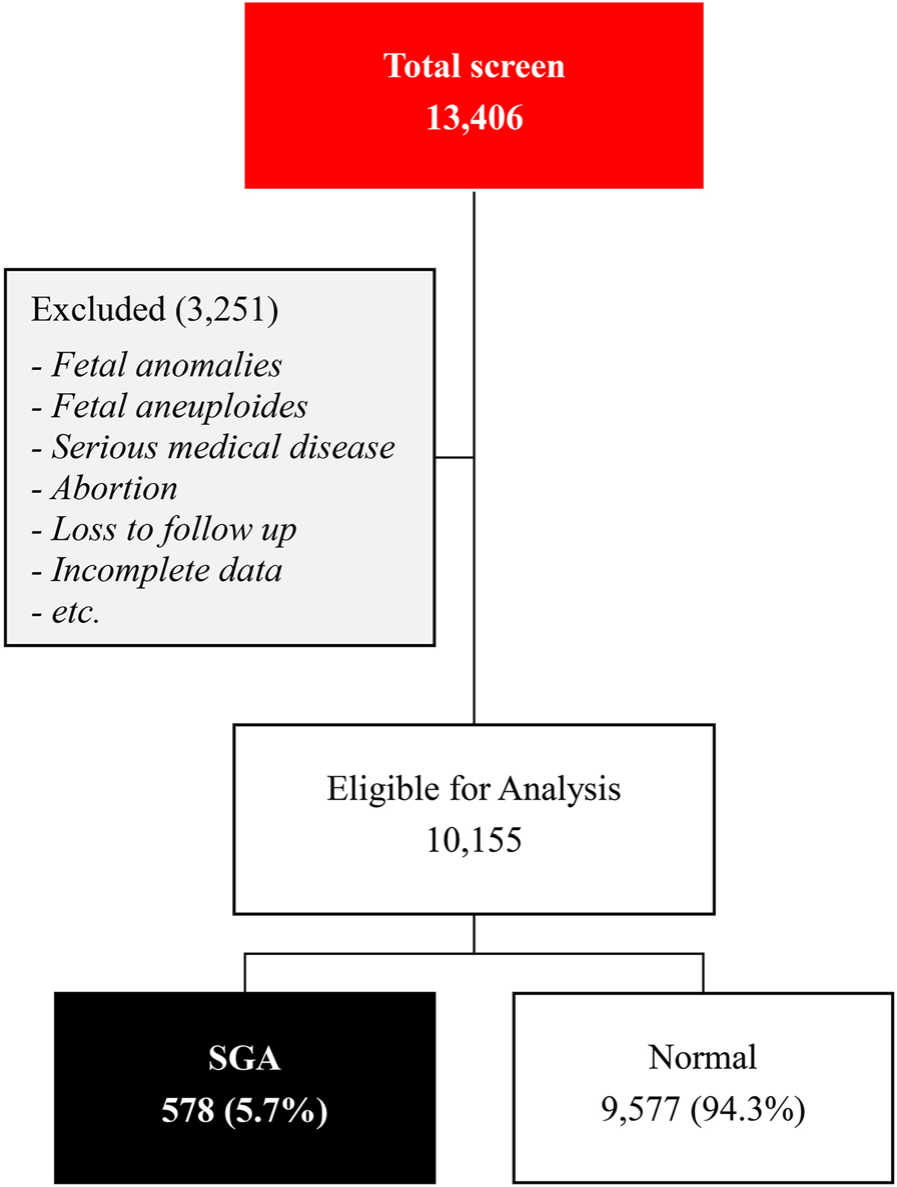
Study flow chart

During the study period of our project of prenatal screening for fetal Down syndrome, 13,406 women underwent quad test (AFP, b-hCG, uE3 and IHA). Among them, 3251 cases were excluded because of various reasons, as presented in Fig. 1, and the remaining 10,155 pregnancies were available for analysis, including 9577 (94.3%) pregnancies with non-SGA and 578 (5.7%) pregnancies with SGA. The baseline characteristics of the two groups are presented in Table 1. Based on multiple regression analyses of the serum biomarker levels (AFP, b-hCG, uE3 and IHA), the fitted models for the expected levels of the four serum biomarkers were constructed, as shown in Table 2**.**

**Table 1:** Baseline characteristics of the pregnant women according to fetal growth status

Characteristics	Non-SGA Group	SGA Group	***P***-value*
Maternal age (yr) + SD	28.3 + 11.2	28.8 + 6.3	0.464
Maternal weight (Kg) + SD	57.5 + 11.3	53.9 + 11.2	< 0.001
Gestational week at sampling +SD	15.5 + 2.0	15.5 + 2.9	0.824
Gestational week at delivery +SD	38.4 + 1.7	37.4 + 2.8	< 0.001
Birth weight (gm) + SD	3068 + 444	2162 + 418	< 0.001
Parity			0.129
•Nulliparous	48.7%	51.9%	
•Parous	51.3%	48.1%	

*Student T test for continuous data, Chi-square test for categorical data

**Table 2:** The models predicting serum biomarker levels

Biomarkers	The models
**AFP levels**	ln (AFP level) = 1.536 + 0.0161 (GA: week) - 0.0054 (Weight: Kg) - 0.0293 (DM); (*r* = 0.368; *p*-value < 0.001)
**b-hCG levels**	ln (b-hCG level) = 1.875–0.0164 (GA: week) - 0.0052 (Weight: Kg) + 0.0006 (Age, year) - 0.0487 (DM); (*r* = 0.392; *p*-value < 0.001)
**uE3 levels**	ln (uE3 level) = 0.2610 + 0.0236 (GA: week) - 0.0013 (Weight: Kg) – 0.0345 (DM) - 0.0005 (Age, year); (*r* = 0.274; *p*-value < 0.001)
**IHA levels**	ln (IHA level) = 2.6962–0.0068 (GA: week) - 0.0032 (Weight: Kg) + 0.0007 (Age, year)- 0.1383(DM); (*r* = 0.398; *p*-value < 0.001)

*DM* diabetes mellitus

**Fig. 2:**
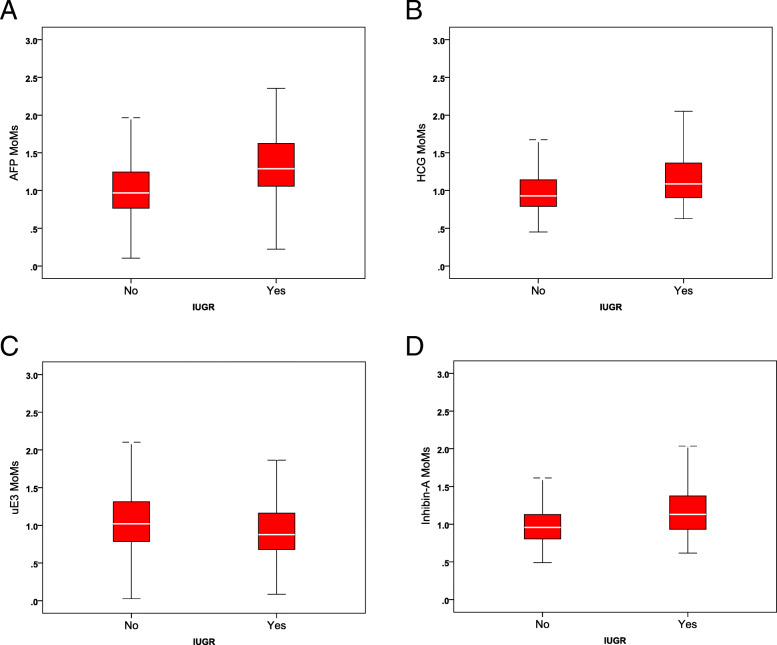
Comparison of AFP MoMs (**a**), b-hCG MoMs (**b**), uE3 (**c**) and hCG (D) between SGA and non-SGA group

Notably, all four serum biomarkers are significantly associated with gestational age, maternal age, maternal weight, and DM status, except that maternal age is not significantly associated with AFP levels. The means and medians of the MoMs of AFP, b-hCG and IHA are significantly higher in the group of SGA than in the non-SGA group, whereas the mean and median of the MoMs of uE3 are significantly lower in the SGA group, as shown in Table 3 and Fig. 2.

**Table 3:** Comparisons of mean and median MoMs of AFP, b-hCG, uE3 and IHA between non-SGA vs SGA group

Group	Non-SGA Group	SGA Group	***P***-value
Means + SD^a^			
AFP MoMs	1.077 + 0.736	1.417 + 0.706	< 0.001
b-hCG MoMs	1.192 + 4.306	1.288 + 0.761	0.590
uE3 MoMs	1.105 + 0.998	0.965 + 0.418	0.001
IHA MoMs	1.046 + 0.443	1.299 + 0.900	< 0.001
Median (IQR)^b^			
AFP MoMs	0.969 (0.48)	1.288 (0.57)	< 0.001
b-hCG MoMs	0.928 (0.35)	1.087 (0.46)	< 0.001
uE3 MoMs	1.020 (0.53)	0.877 (0.34)	< 0.001
IHA MoMs	0.957 (0.32)	1.129 (0.45)	< 0.001

*IQR* interquartile range

^a^Student T test

^b^Mann-Whitney-U test

In the categorization of serum biomarkers into normal and abnormal levels, conventional serum biomarker cutoffs [21] (> 2 MoM for AFP, b-hCG, and IHA, and <  0.5 MoM for uE3) were used to predict SGA. All four biomarkers were significantly associated with the rate of SGA. Abnormal AFP MoM had the strongest predictivity with a relative risk of 2.81 (95% CI: 2.20–3.58), followed by b-hCGMoM, IHA MoM, and uE3 MoM, respectively, as presented in Table 4.

**Table 4:** Relative risk of abnormal serum biomarkers MoMs of AFP, b-hCG, uE3 and IHA between non-SGA vs SGA group

Group	Non-SGA Group (n: 9577)	SGA Group (n: 578)	***P***-value	Relative risk (95%CI)
AFP > 2 MoMs	355 (85.1%)	62 (14.9%)	< 0.001	2.81 (2.20–3.58)
AFP < 2 MoMs	9222 (94.7%)	516 (5.3%)		
b-hCG > 2 MoMs	321 (85.4%)	55 (14.6%)	< 0.001	2.74 (2.11–3.54)
b-hCG < 2 MoMs	9256 (94.7%)	523 (5.3%)		
uE3 < 0.5 MoMs	316 (90.8%)	32 (9.2%)	0.004	1.65 (1.17–2.32)
uE3 > 0.5 MoMs	9261 (94.4%)	546 (5.6%)		
IHA > 2 MoMs	253 (88.5%)	33 (11.5%)	< 0.001	2.09 (1.50–2.91)
IHA < 2 MoMs	9324 (94.5%)	545 (5.5%)		

**Fig. 3:**
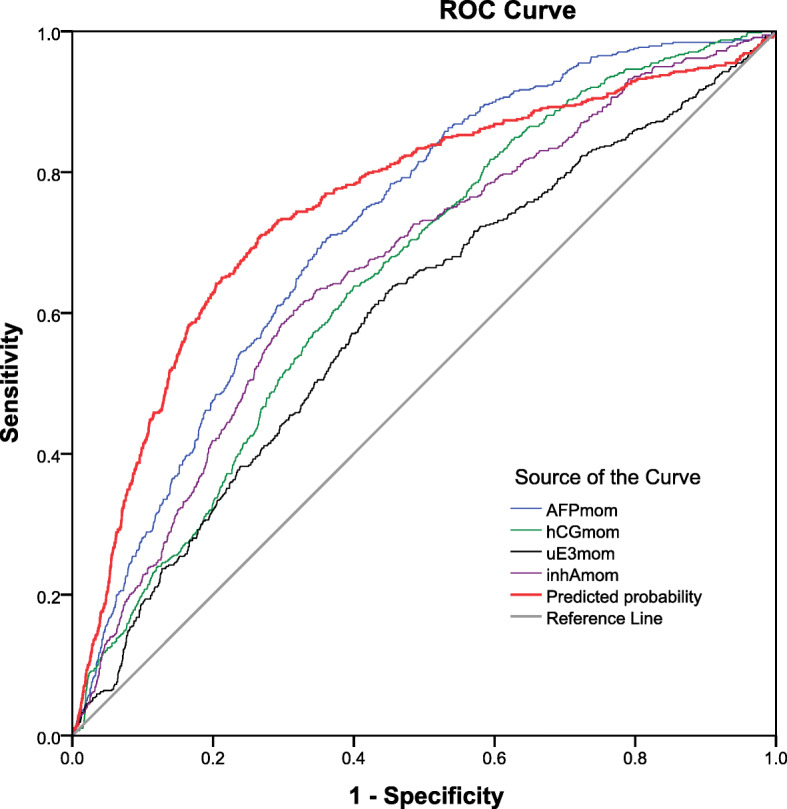
ROC curves shows performance in predicting small-for-gestational age fetuses of the individual serum biomarker levels and their combination. Area under ROC Curve. MoMs of AFP: 0.724 (95%CI: 0.705–0.743). MoMs of b-hCG: 0.655 (95%CI: 0.634–0.676) MoMs of uE3: 0.597 (95%CI: 0.573–0.621). MoMs of IHA: 0.664 (95%CI: 0.642–0.687). Combined of serum biomarkers (predicted probability): 0.754 (95%CI: 0.732–0.777).

**Fig. 4:**
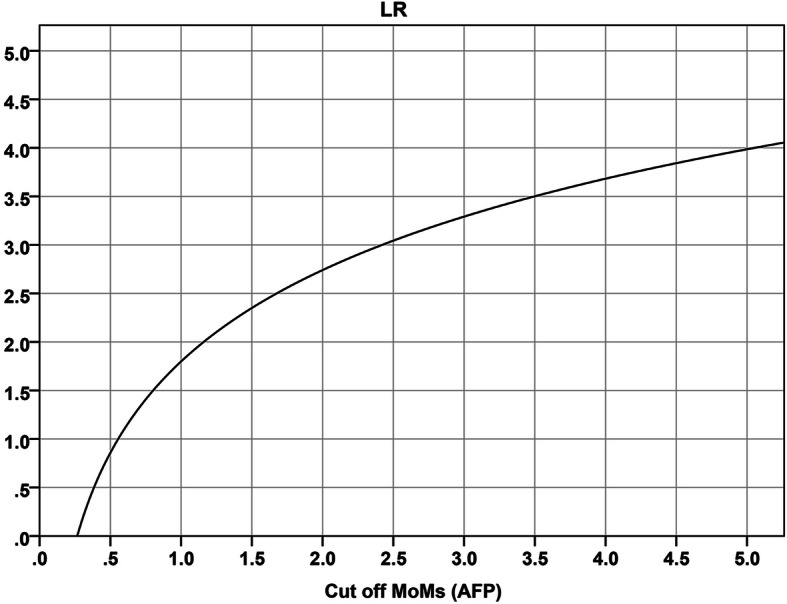
Likelihood ratio for small-for-gestational age fetuses, based on MoMs of AFP

Using binary logistic regression analysis, the predictive models for SGA involving each of the four serum biomarkers and their combination were constructed, as shown in Table 5. Note that the combination does not contain b-hCG MoM because the addition of b-hCG MoM to the equation has no significant additive diagnostic value, though b-hCG MoM has diagnostic value when used as an individual biomarker. Based on the constructed models, the diagnostic performances of the individual biomarkers and their combination in the prediction of SGA are analyzed using ROC (receiver-operated characteristics) curve, as presented in Fig. 3. The combination of the serum biomarkers gives the highest predictivity, with an area under curve of 0.754 (95%CI: 0.732–0.777). Note that for the individual serum markers, AFP MoM has the highest predictive value, giving an area under curve of 0.724 for SGA, followed by IHA MoM. The higher the MoM of AFP, the higher the likelihood of SGA, as presented in Fig. 4.

**Table 5:** Probability of SGA based on serum individual biomarker level and their combination

Biomarkers	The models predicting small-for-gestational age (probability)
**AFP MoMs**	ln (Odds ratio) = −3.1403 + 0.2863 (AFP MoM)
**b-hCG MoMs**	ln (Odds ratio) = −2.8127 - 0.0042 (b-hCG MoM)
**uE3 MoMs**	ln (Odds ratio) = − 2.0289 - 0.7609 (uE3 MoM)
**IHA MoMs**	ln (Odds ratio) = − 3.4347 - 0.5558 (b-hCG MoM)
**Combined biomarkers**	ln (Odds ratio) = −4.4908 + 1.6877 (AFP MoM) + 0.8965 (IHA MoM) - 0.3248 (AFP MoM x IHA MoM) - 0.6725 (AFP MoM x uE3 MoM)

### An example of prediction of SGA based on quad test

A 34-year old pregnant woman underwent quad test at 15 weeks of gestation, which revealed the following: AFP level: 13.80 U/mL; hCG level: 22.89 ng/mL; uE3 level: 3.09 nmol/L; IHA level: 2082.54 pg/mL. The patient had no pre-gestational DM, and her body weight was 60 Kg.

Based on the models in Table 2 and the baseline data of maternal age, gestational age, DM status and body weight, the MoMs of the four biomarkers of this woman are as follows: AFP = 0.43 MoM; hCG = 0.92 MoM; uE3 = 0.93MoM; IHA = 7.18 MoM.

By substituting the specific MoM of each biomarker above into the combined biomarker model in Table 5, the resulting log (Odds ratio) = 0.80223, converted to Odds ratio: 6.34. Therefore, this pregnancy is at a very high risk of SGA.

## Discussion

Insights gained from this study include the following: 1) Fetuses with growth restriction have significantly higher MoMs of AFP, b-hCG and IHA but lower MoMs of uE3. 2) Abnormal MoMs of all individual biomarkers based on the conventional MoM cutoffs are significantly associated with an increased risk of SGA. AFP has the strongest association, but they all have significant predictive values for SGA. 3) The predictive models of each biomarker and their combination are constructed. The combined model yields the best prediction. 4) The combination of all biomarkers increases the predictive power. However, b-hCG has no additive value in the combination. 5) If the combined model is integrated into the built-in software of the machine, the risks of SGA and Down syndrome could be simultaneously estimated and reported using the same set of serum biomarkers.

The associations between unexplained abnormal levels of serum biomarkers and fetal Down syndrome / SGA have been published several times. Most studies focused on elevated AFP levels. Studies that included all four biomarkers are rare [21, 22]. Importantly, most previous studies reported the correlation between individual biomarkers and poor outcomes, whereas the effectiveness of their combination is rarely described. Moreover, most previous studies used arbitrary cutoff to define abnormal MoMs, while the risk is quantitatively level-dependent rather than the all-or-none fashion. To the best of our knowledge, only Odibo et al. [23] evaluated the optimal thresholds of unexplained abnormal triple biomarkers to predict SGA by ROC curve, but they did not include IHA, did not evaluate the combination, and the sample size was relatively small. Differently, our study assessed the effectiveness of both individual biomarkers and their combination and constructed the models that may be used to estimate the overall risk of SGA for each patient as well as simply used in actual practice. The models might seem complicated, but they can easily be integrated into a computerized calculator to automatically report once the measured biomarker levels are input. However, our findings should be interpreted with caution. The results are derived specifically from Thai population and might not be reproducible in other groups with different demography. Note that the prevalence of SGA in this study was 5.7%, much lower than theoretically expected (10%). The reason is unclear, but it might be due to the use of inappropriate reference ranges in defining SGA [24]. Also, it is possibly due to the fact that pregnancies with higher risk of SGA were excluded, making the overall incidence relatively low.

The strengths of this study are as follows: 1) Our dataset was prospectively collected, and the participants were followed-up for final obstetric and neonatal outcomes by the project team. 2) Because of its population-based nature, the constructed models are more likely generalizable. 3) The sample size was adequate for comparison of SGA rate. 4) Because of the high homogeneity of the study population, consisting of only Thai pregnant women, the results were not confounded by racial factor. 5) The MoMs of each biomarker were derived from our own population, not automatically calculated from the built-in models, which are based on Caucasian women. Thus, it is more suitable to apply our models to Thai or Asian women. 6) Our predictive models can be used to estimate the individual risk of each woman as a quantified risk, or the individual likelihood ratio, instead of using one arbitrary cut-off MoM value to categorize risk as just low or high, as reported in most previously published studies.7) The predictive models can be integrated into the built-in program for an automatic calculation of the fetal risk of SGA in the same manner of predicting the risk of Down syndrome. The same set of serum biomarkers could be simultaneously used to estimate the risks of Down syndrome and SGA, without extra cost and extra effort.

The limitations of this study include the following points: 1) The study did not take other risk factors of SGA into account. Some potential risk factors of SGA, such as smoking habit, alcohol use, and underlying diseases (like chronic hypertension and antiphospholipid syndrome), were not incorporated into the analysis. However, our models focused on the performances of the biomarkers in predicting the overall risk of SGA regardless of any preexisting risk factors. 2) The predictive models may not be properly applied to other populations with different ethnicity and demographic data. Nevertheless, this study confirmed the association between abnormal levels of the four biomarkers and an increased risk of SGA. Our findings may encourage researchers in other ethnic groups to create the models specific for their own population. 3) A large number of women were excluded from analysis because of unavailability of final outcomes or loss to follow-up. The difference in baseline characteristics between the excluded and included groups is not known. 4) Bias in patient classification might have existed, since the prevalence of SGA was somewhat lower than expected, which should be 10% as its definition. Nevertheless, this might be partly explained by the exclusion of the cases with higher risk of SGA because of underlying diseases, such as chronic hypertension, heart disease, thyroid diseases, etc. 5) The accuracy of the predictive model has not been assessed by a validation cohort. Thus, further studies testing its reproducibility should be performed before clinical application.

## Conclusion

This study suggests that the daily used quad test can also be used as a screening method for fetal SGA. All four biomarkers are significantly associated with SGA. Regarding individual biomarkers, AFP has the strongest predictive power. The highest predictive value is derived from their combination. However, incorporation of b-hCG levels into the combined model had no additive value. We could simply take advantage of quad test by integrating the combined model for SGA into the built-in software for Down syndrome screening to estimate the risks of SGA and Down syndrome in the same report. Nevertheless, this study is preliminary, and further studies on other ethnic groups should be undertaken to evaluate the reproducibility.

## Data Availability

The datasets analyzed during the current study are available from the corresponding author upon reasonable request.

## Abbreviations

AFP: Alpha-fetoprotein
SGA: Small-for-gestational age
hCG: Beta-human gonadotropin
IHA: Inhibin-A
MoM: Multiple of medians
uE3: Unconjugated estriol
